# Association between cerebral atrophy and osteoporotic vertebral compression fractures

**DOI:** 10.1371/journal.pone.0224439

**Published:** 2019-11-05

**Authors:** In-Suk Bae, Jae Min Kim, Jin Hwan Cheong, Myung-Hoon Han, Je Il Ryu

**Affiliations:** Department of Neurosurgery, Hanyang University Guri Hospital, Guri, Gyonggi-do, Korea; Medical College of Wisconsin, UNITED STATES

## Abstract

**Purpose:**

Osteoporotic vertebral compression fractures (OVCFs) have a serious impact on people’s health and quality of life. The purpose of this study was to analyze brain volume in patients with osteoporosis using brain magnetic resonance imaging (MRI) and to investigate the relationship with osteoporotic vertebral compression fractures.

**Materials and methods:**

We included 246 patients with osteoporosis who underwent thoracolumbar radiographs and brain MRI at our hospital. Clinical data on age, sex, bone mineral density, height, weight, osteoporosis medication, hypertension, diabetes, alcohol drinking, and smoking were collected. Intracranial cavity, brain parenchyma, and lateral ventricles volumes were measured using brain MRI with a semiautomated tool.

**Results:**

We founded an independent correlation between age and volume percentages of the brain parenchyma and lateral ventricles. We observed a statistically significant decrease in volume percentage of the brain parenchyma and an increase in volume percentage of the lateral ventricles with increasing age. In addition, we confirmed that patients with OVCF showed a significantly lower volume percentage of brain parenchyma than patients without OVCF.

**Conclusion:**

We observed a significant association between OVCF and volume percentage of brain parenchyma. Degeneration of the brain may lead to a high incidence of falls, and OVCF may occur more frequently in patients with osteoporosis.

## Introduction

Osteoporosis is a degenerative disorder with compromised bone strength that increases the risk of fracture. Osteoporosis is often seen in the elderly population, and osteoporotic fracture leads to high rates of morbidity and mortality in old age [1,2]. Osteoporotic vertebral compression fractures (OVCFs) are the third most common fracture in the world, with an estimated annual incidence of new OVCF of 1.4 million [3]. Osteoporosis and related fragility fractures have a serious impact on people’s health and quality of life because they can result in chronic pain, morbidity, and mortality.

Approximately 1–5% of all fractures result from falls in the elderly [4]. More than one-third of patients aged 65 years and above experience an episode of fall, and half of these have repeated falls [5,6]. Several studies have attempted to correlate the cause of fall with brain degeneration. Moreover, degeneration of the frontal lobes, basal ganglia, or cerebellum as well as hydrocephalus have been proposed as possible brain lesions in groups prone to falls [7,8].

Cerebral atrophy and ventricular enlargement that occur during the normal aging process are known to lead to dementia and loss of cognitive functions [9]. For decades, dementia has been acknowledged as a major risk factor for fall and bone fracture. Numerous cross-sectional imaging studies have found correlation between increasing age and decreasing brain volumes [10,11]. However, there is no study regarding the relationship between OVCF and brain volumes of ventricles and parenchyma.

The purpose of this study was to analyze the intracranial cavity volume (ICV), brain parenchymal volume (BPV), and lateral ventricles volume (LVV) in patients with osteoporosis using brain magnetic resonance imaging (MRI) and to investigate the relationship with OVCFs in these patients.

## Materials and methods

### Study design

We retrospectively recruited patients with osteoporosis who underwent brain MRI and thoracolumbar radiographs at least once in our hospital from January 1, 2008 to December 31, 2018. We initially identified 1,526 patients whose records indicated one or more procedure codes for thoracolumbar radiograph and brain MRI.

Patients were eligible for inclusion in this study if they met the following criteria (1) age ≥ 65 years; (2) T-score ≤ −2.5; (3) procedure codes for thoracolumbar radiograph and brain MRI; and (4) brain MRI procedure performed before thoracolumbar radiograph.

**Fig 1:**
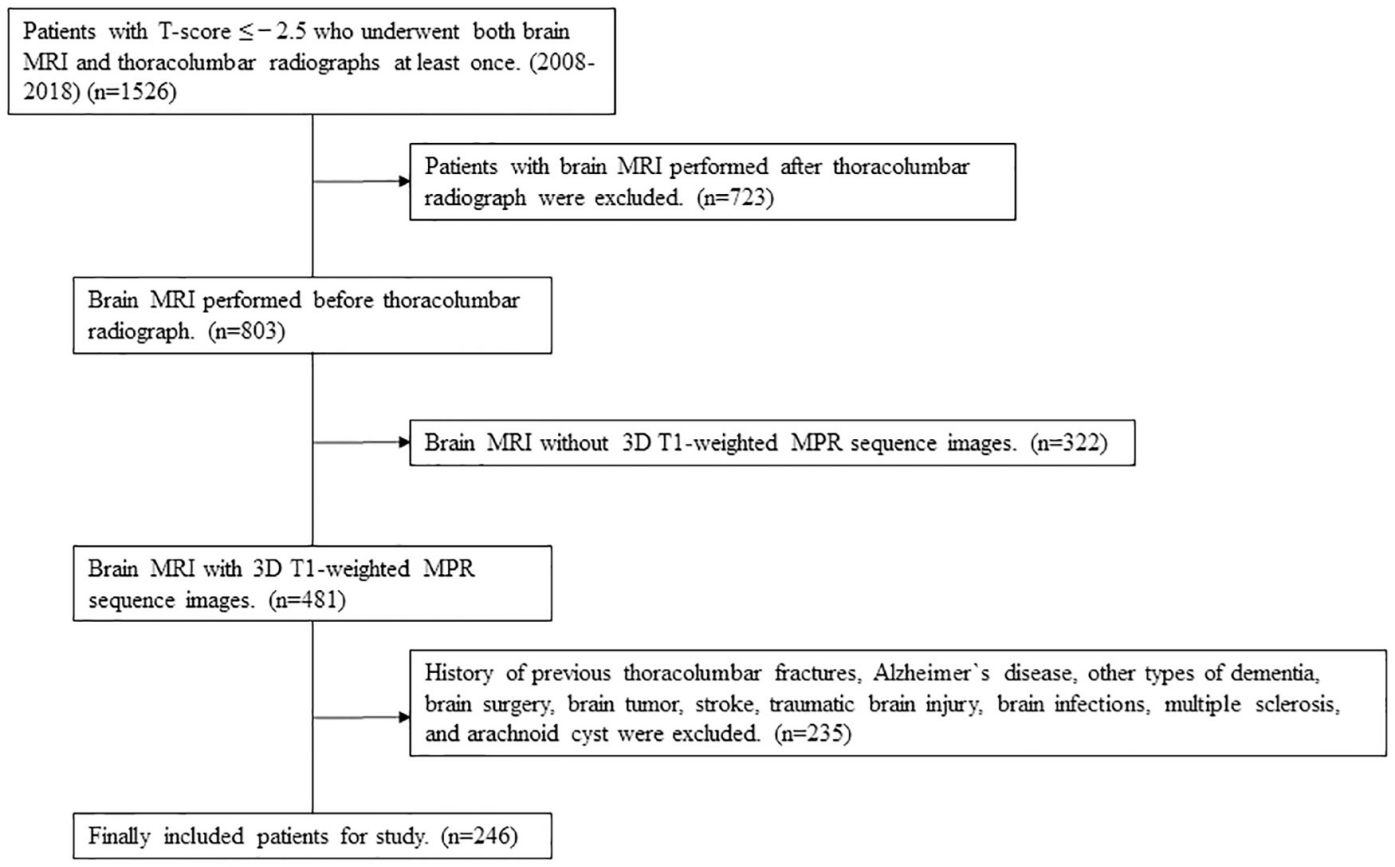
Flow chart for selecting patients who underwent both brain MRI and thoracolumbar radiographs in our hospital. MRI = magnetic resonance imaging; MPR = multiplanar reconstruction.

Exclusion criteria were the following: (1) pathologic fracture due to factors such as malignancy; (2) a history of major trauma (minimal trauma fracture, defined as a fracture after a fall from standing height were included); (3) patients in whom a brain MRI did not include T1-weighted axial multiplanar reconstruction (MPR) sequence (3-mm-thick slices); (4) a history of previous thoracolumbar fractures; and (5) a history of Alzheimer`s disease (AD), other types of dementia, brain surgery, brain tumor, stroke (ischemic, hemorrhagic), traumatic brain injury, and arachnoid cyst. Ultimately, 246 patients were included in the study (Fig 1).

This study was approved by the Institutional Review Board of Hanyang University Guri Hospital, Korea. Owing to the retrospective nature of the study, the need for informed consent was waived by the IRB of our institution. All patient records were anonymized prior to analysis.

### Bone mineral density measurement

Bone mineral density (BMD) information of the patients was investigated retrospectively by reviewing charts of Dual-energy X-ray absorptiometry (DXA). DXA to assess the BMD (g/cm^2^) of the lumbar spine L1–L4 and femoral neck was performed using a Discovery Wi DXA system (Hologic, Bedford, MA). Each BMD value was converted into a T-score. T-score reference ranges were calculated using data provided by the bone densitometry equipment manufacturer from healthy young Asian women. The lower T-score value between the lumbar spine and femoral neck was adopted as the T-score for the study. Based on the World Health Organization T-score classification, osteoporosis was defined as a T-score ≤ −2.5.

### Thoracolumbar radiograph assessment

Thoracolumbar radiographs consisted of anteroposterior and lateral spinal radiographs performed at diagnosis. The compression ratio of the fractured vertebral body was calculated on the lateral radiograph using the following equation: Compression ratio (%) = (1-fractured height / estimated prefractured height) x 100. The estimated prefractured height was measured as the mean value after summing the vertebral body height above and below the fractured vertebral body.

### Brain MRI image acquisition

All 3D T1-weighted MPR MR images (slice thicknesses, 1.5–3.0 mm) were obtained using the Ingenia 3.0 Tesla CX (Philips Ingenia, Philips Medical Systems, Böblingen, Germany) and Achieva 3.0 Tesla TX (Philips Achieva, Philips Medical Systems, Böblingen, Germany) scanners at our hospital. A previous study described that MPR sequences with a 3.0 Tesla MRI provided excellent visualization for the inner structures of the head and brain [12].

### Volumetric assessment of intracranial cavity, brain parenchyma, and lateral ventricles

We measured ICV, BPV, and LVV with updated 3D slicer software from an open-source medical image–computing platform, version 4.10.1. (http://www.slicer.org). The reliability of the 3D slicer has been described elsewhere, including detailed descriptions of the slicer’s various functions [13,14]. All procedures were performed by a well-trained 3D-slicer user (I.S.B). The T1-weighted MR images were used for analysis in all study patients, as T1-weighted MR imaging is appropriate for skull stripping (https://www.slicer.org/wiki/Modules:SkullStripperModule) and measurement of brain parenchyma and CSF space volumes using a slicer [15]. In addition, as the T1 MPR sequence with the 3.0 Tesla MRI in our hospital showed a relatively thin slice thickness of 1.5–3.0 mm, we were able to perform accurate volumetric measurements.

**Fig 2:**
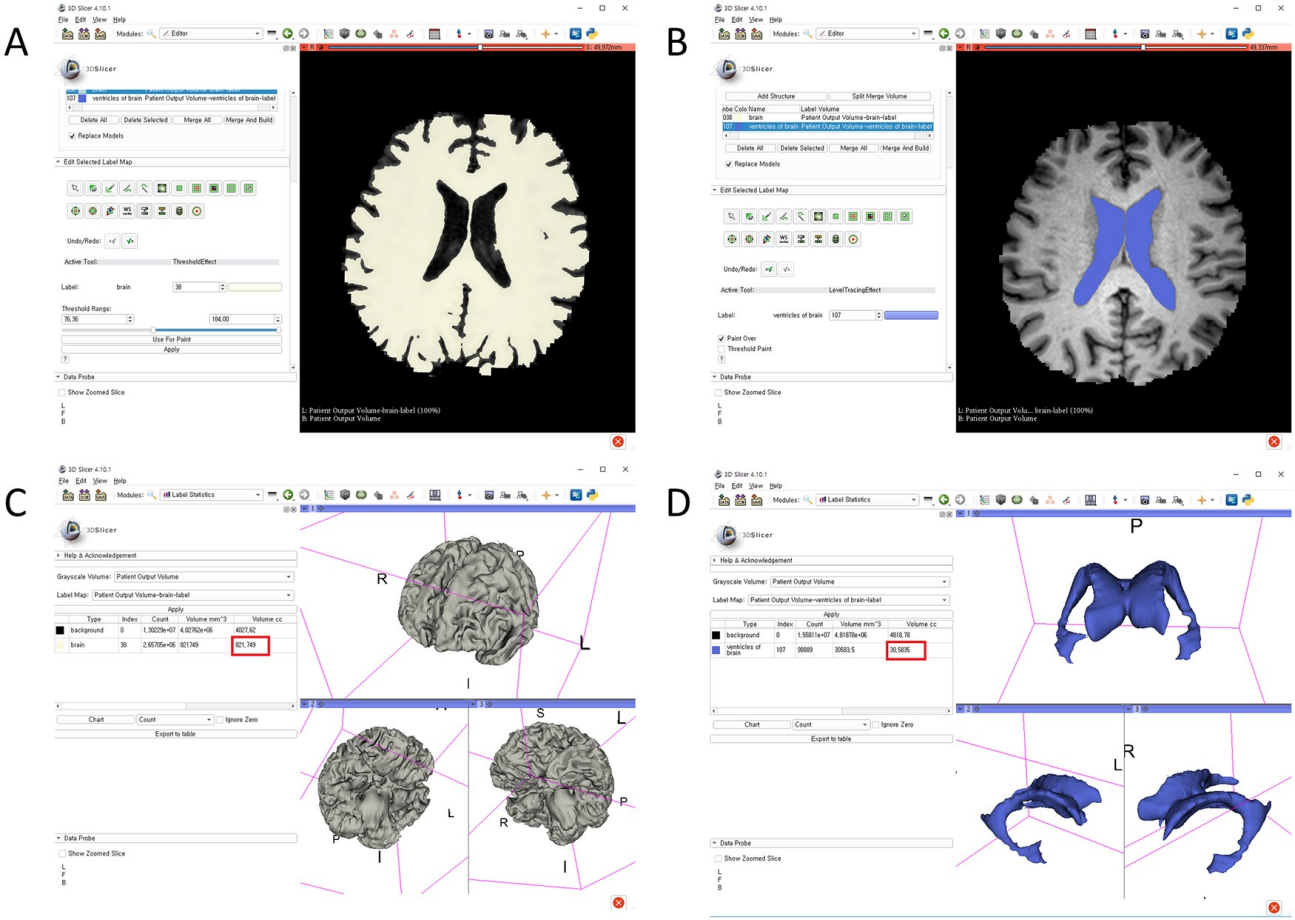
Segmentation of the brain parenchyma and lateral ventricles with a 3D-reconstructed model using the 3D slicer and calculation of each volume (red box indicates the volume): Threshold-based methods to segment the (A) brain parenchyma and (B) lateral ventricles; (C) 3D reconstruction of brain parenchyma using the Model Maker function; (D) 3D reconstruction of lateral ventricles using the Model Maker function.

**Fig 3:**
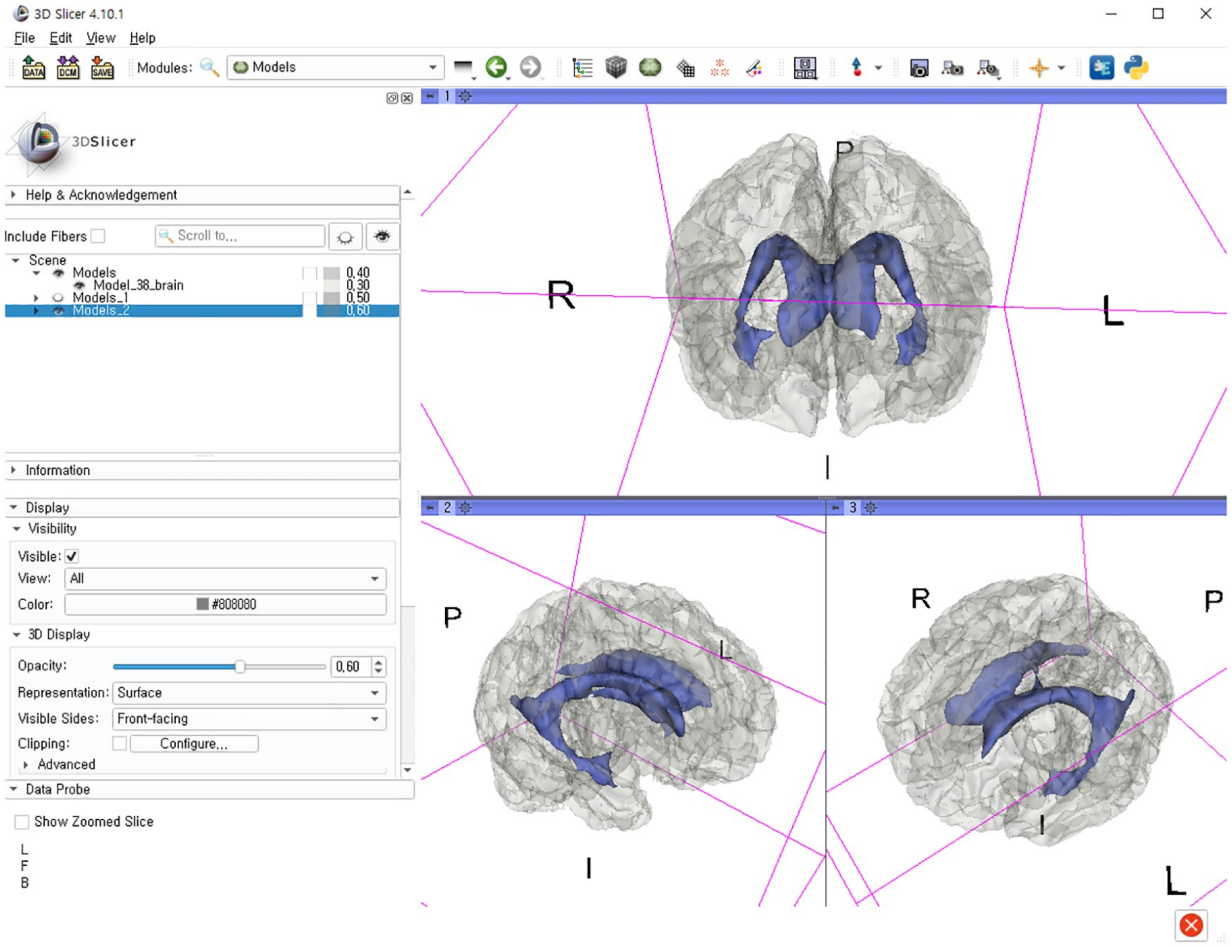
Merged brain parenchyma and lateral ventricles after 3D-reconstructed model using the 3D slicer.

The stepwise methods of volumetric assessment using the 3D slicer were as follows: (1) brain MRI DICOM files from the picture archiving and communication system (PACS) were loaded to the software; (2) the Swiss Skull Stripper function was used to strip the skull from the loaded MRI to segment intracranial cavity; (3) threshold-based methods were then used to segment the brain parenchyma and lateral ventricles; (4) 3D reconstruction was performed using the Model Maker function; and (5) the Label Statistics function was used to calculate ICV, BPV, and LVV (Figs 2 and 3).

### Medical variables

Medical information of the patients was investigated using medical charts. Clinical data on age, sex, height, weight, osteoporosis medication, hypertension, diabetes, alcohol drinking, and smoking were collected. BMI was calculated as weight/(height × height) and expressed in kg/m^2^.

### Statistical methods

Continuous variables are expressed as mean ± standard deviation (SD) or median with interquartile range, while discrete variables are expressed as count and percentage. The chi-square test and Student’s *t*-test were used to assess clinical differences between variables in patients with or without OVCF.

The volume percentages of brain parenchyma and lateral ventricles were calculated as (BPV/ICV)×100% and (LVV/ICV)×100%, respectively. Multivariable linear regression was performed to identify independent associations between OVCFs and the intracranial cavity volume percentages of the brain parenchyma and lateral ventricles. Variables with a p-value < 0.10 in univariate analysis were entered in a multivariable logistic regression model using a backward stepwise method. Odds ratios (ORs) and 95% confidence intervals (CI) were reported for statistically significant factors (p-value < 0.05).

Box plots with dot plots were used to visualize the association between the volume percentages of brain parenchyma and lateral ventricles in patients with or without OVCF. P-values less than 0.05 were considered statistically significant. All statistical analyses were performed using R version 3.5.2 (https://www.r-project.org/; R Foundation for Statistical Computing, Vienna, Austria).

## Results

### Demographic Characteristics of patients

We enrolled 246 patients with osteoporosis who underwent brain MRI and thoracolumbar radiographs at least once with no history of Alzheimer`s disease (AD), other types of dementia, brain surgery, brain tumor, stroke, traumatic brain injury, brain infections, or arachnoid cysts. The average age of the patients was 72.6 years, and 87.0% of patients were women. A total of 109 patients had OVCFs. The median bone mineral density (BMD) was -2.94. The mean ICV, BPV, and LVV were 1216.6 cc, 1011.5 cc, and 31.8 cc, respectively. The mean volume percentages of the brain parenchyma and lateral ventricles to the intracranial cavity were 83.1% and 2.6%, respectively. We found significant differences in age, BPV, and percentages of the brain parenchyma between patients with OVCFs and those without OVCFs. Further descriptive data of study patients are shown in Table 1.

**Table 1:** Characteristics of study patients.

Characteristics	Vertebral compression fracture (-)	Vertebral compression fracture (+)	Total	P
Number	137	109	246	
Sex, female, n (%)	122 (89.1)	92 (84.4)	214 (87.0)	0.376
Age, mean ± SD, y	70.5 ± 6.0	75.3 ± 6.1	72.6 ± 6.5	<0.001
ICV, mean ± SD, cc	1222.9 ± 91.3	1208.8 ± 77.4	1216.6 ± 85.6	0.198
BPV, mean ± SD, cc	1036.9 ± 95.5	979.5 ± 87.2	1011.5 ± 95.1	< 0.001
LVV, mean ± SD, cc	28.8 ± 14.6	35.5 ± 17.8	31.8 ± 16.4	0.002
Volume percentage of the brain parenchyma, mean ± SD, %	84.8 ± 4.2	81.0 ± 3.8	83.1 ± 4.4	< 0.001
Volume percentage of the lateral ventricles, mean ± SD, %	2.4 ± 1.2	3.0 ± 1.5	2.6 ± 1.4	0.001
T-score, mean ± SD	-2.9 ± 0.4	-3.0 ± 0.4	-2.94 ± 0.5	0.011
BMI, mean ± SD, kg/m^2^	23.6 ± 3.4	23.4 ± 3.9	23.4 ± 3.8	0.631
Height, mean ± SD, m	1.55 ± 0.08	1.53 ± 0.07	1.54 ± 0.08	0.032
Weight, mean ± SD, kg	56.7 ± 9.8	54.5 ± 8.8	55.7 ± 9.4	0.066
Osteoporosis medication (%)	67 (48.9)	49 (45.0)	116 (47.2)	0.625
Hypertension, n (%)	50 (36.5)	56 (51.4)	106 (43.1)	0.027
Diabetes, n (%)	39 (28.5)	27 (24.8)	66 (26.8)	0.613
Alcohol, n (%)	17 (12.4)	16 (14.7)	33 (13.4)	0.741
Smoking, n (%)	7 (5.1)	7 (6.4)	14 (5.7)	0.869

SD, standard deviation; ICV, intracranial cavity volume; BPV, brain parenchymal volume; LVV, lateral ventricles volume; BMI, body mass index

### Association between age and volume percentages of the brain parenchyma and lateral ventricles to the intracranial cavity

**Fig 4:**
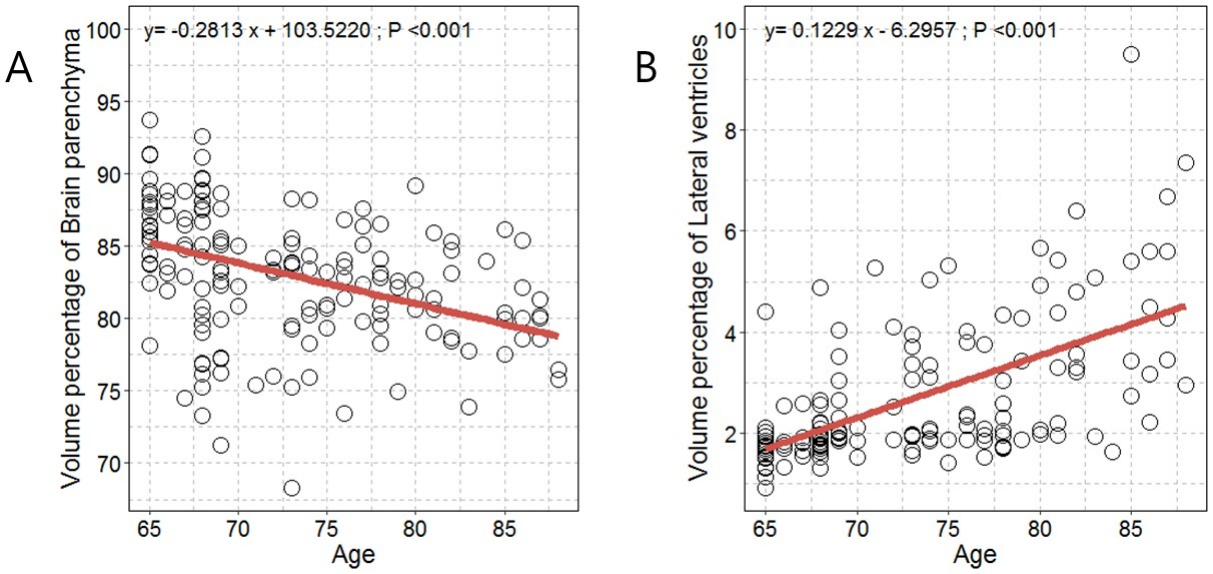
Scatterplot with linear regression line showing the associations between age and volume percentages of (A) brain parenchyma and (B) lateral ventricles.

Significant positive and negative correlations were identified between age and volume percentages of the brain parenchyma and lateral ventricles (Fig 4). We observed a statistically significant decrease in volume percentage of the brain parenchyma with increasing age (Fig 3A). We observed a statistically significant increase in volume percentage of the lateral ventricles with increasing age (Fig 3B). After adjusting for all covariates, we observed an independent correlation between age and volume percentages of the brain parenchyma and lateral ventricles (P<0.001).

### Association between brain volumes and osteoporotic vertebral compression fractures

Univariate analysis showed that the odds ratio for OVCF increased with age and low volume percentages of brain parenchyma. A logistic regression analysis of OVCFs is shown in Table 2. Two variables remained statistically significant after adjusting for confounding factors: older age (OR 1.09, 95% CI = 1.03–1.14, p = 0.0011) and higher volume percentages of brain parenchyma (OR 0.21, 95% CI = 0.07–0.66, p = 0.0073). We confirmed that patients with OVCF showed significantly lower volume percentage of brain parenchyma.

**Table 2:** Logistic regression analysis for present of osteoporotic vertebral compression fracture.

	Univariate		Mulivariable	
Variable	OR (95% CI)	P value	OR (95% CI)	P value
Sex	1.01 (0.35–2.94)	0.9880		
Age	1.10 (1.03–1.17)	0.0049	1.09 (1.03–1.14)	0.0011
ICV	0.91 (0.83–0.99)	0.0329	0.91 (0.95–0.98)	0.0185
BPV	1.12 (1.01–1.25)	0.0293	1.12 (1.02–1.22)	0.0177
LVV	1.05 (0.77–1.43)	0.7501		
Volume percentage of the brain parenchyma	0.19 (0.05–0.71)	0.0134	0.21 (0.07–0.66)	0.0073
Volume percentage of the lateral ventricles	0.44 (0.01–1.93)	0.6704		
T-score	0.81 (0.38–1.77)	0.6039		
BMI	0.98 (0.89–1.08)	0.7200		
Osteoporosis medication	0.95 (0.51–1.76)	0.8651		
Hypertension	1.23 (0.62–2.46)	0.5564		
Diabetes	0.53 (0.26–1.11)	0.0908	0.57 (0.28–1.13)	0.1080
Alcohol	1.50 (0.53–4.25)	0.4421		
Smoking	1.13 (0.29–4.46)	0.8583		

ICV, intracranial cavity volume; BPV, brain parenchymal volume; LVV, lateral ventricles volume; CI, confidence interval; MRI, magnetic resonance imaging; BMI, body mass index

### Comparison of volume percentages of brain parenchyma and lateral ventricles according to OVCF

**Fig 5:**
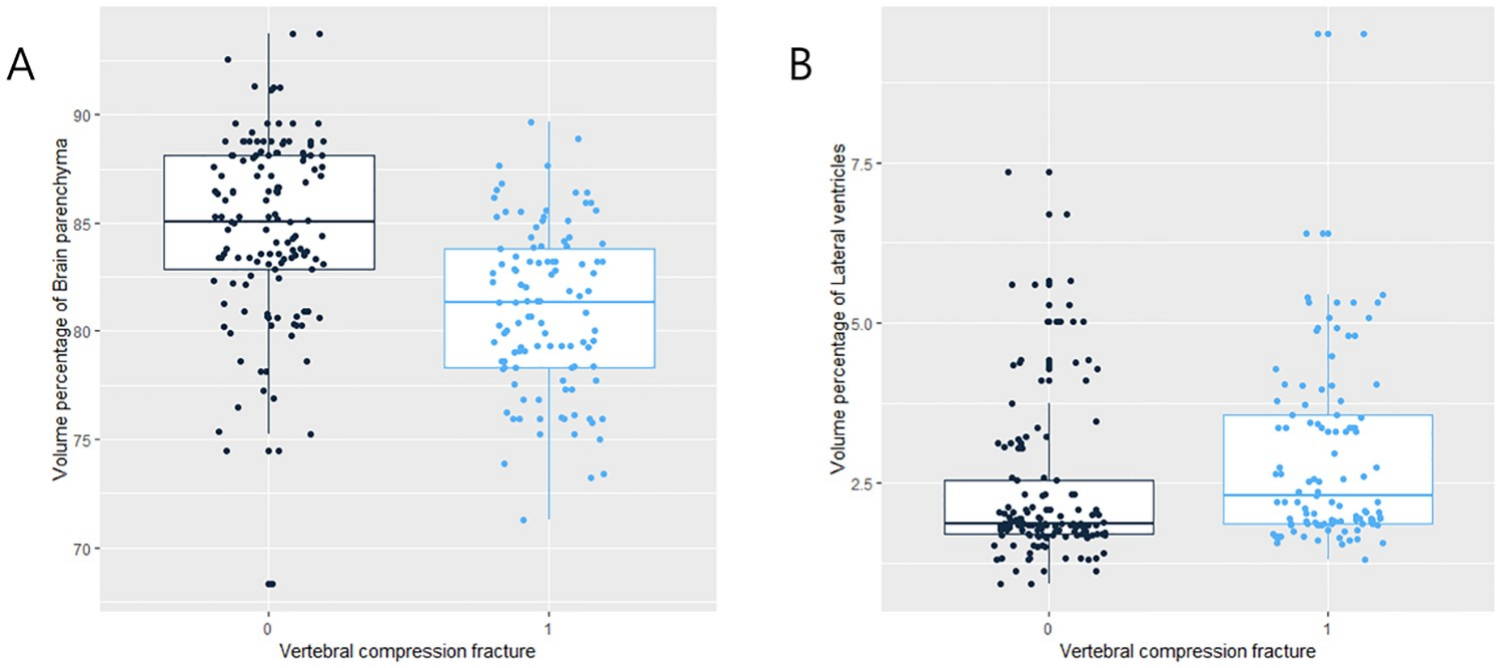
Boxplots with dot plots of the volume percentages of (A) brain parenchyma and (B) lateral ventricles according to OVCF. OVCF = Osteoporotic vertebral compression fracture.

A statistically significant tendency of lower volume percentage of the brain parenchyma was found in patients with OVCF (Fig 5A). Also, we found a higher volume percentage of lateral ventricles in patients with OVCF (Fig 5B). However, there were no significant differences of volume percentage of lateral ventricles between patients with and without OVCFs.

## Discussion

We investigated the relationships between brain volumes and OVCF in patients with osteoporosis. We found an independent correlation between age and volume percentages of the brain parenchyma and lateral ventricles. In the current study, we confirmed that patients with OVCF showed significantly lower volume percentage of brain parenchyma. Conversely, we observed a higher volume percentage of lateral ventricles in patients with OVCF. To the best of our knowledge, this is the first study to suggest that brain volume is related to OVCF.

Both cerebral atrophy and osteoporosis have been reported as common chronic disorders in aging populations. It is well known that physiological brain atrophy and ventricular dilatation accelerate with increasing age [16]. Also, the prevalence of dementia, such as Alzheimer’s disease, increases exponentially after the age of 65 years [17]. Previous studies have shown that patients with Alzheimer’s disease have reduced hip BMD and a nearly 2-fold risk of hip fracture [18,19].

Numerous cross-sectional imaging studies have found a correlation between increasing age and decreasing brain volumes [10,11]. Li et al. reported that cerebral volume reduction was correlated with cognitive deficits [20]. A previous study revealed that patients with Alzheimer’s disease had greater ventricular enlargement than subjects with mild cognitive impairment and normal elderly controls [21]. Both normal aging and Alzheimer’s disease are associated with changes in grey and white matter volumes, and it is hypothesized that Alzheimer’s disease might be correlated with the aging process [22]. Accordingly, we excluded patients with a history of Alzheimer`s disease and other types of dementia to investigate the relationships between OVCF and brain volumes in patients without dementia. In our study, we found lower volume percentage of brain parenchyma and higher volume percentage of lateral ventricles with increasing age.

Masdeu et al. revealed that elderly individuals who experienced fall had a significantly greater degree of white matter hypodensity on computed tomography (CT) [23]. They showed the relation between fall and cerebral lesion. Hypodensity on brain CT appeared to be weaker in parenchymal brain tissue, which indicated decline of brain volume. However, that study used CT images rather than MRI. In our study, we analyzed 3D MRI brain volume, which is a more accurate estimation.

According to the studies mentioned previously, we hypothesized that the aging process of the brain may lead to brain atrophy and ventricular dilatation. This degenerative change to the brain can affect patients by increasing the tendency to fall. Consequently, OVCFs may occur more frequently in patients with parenchymal atrophy and ventricular enlargement.

Previous studies reported about the role of the nervous system on bone modeling [24,25]. In addition, some animal study established that the regulation of bone remodeling could be regulated by the central nervous system [26–29]. Studies have also revealed that there is neurogenic control of bone metabolism through the interplay of central and peripheral nervous system. They proved the existence of a neural arm regulating bone remodeling. The interaction between the bone and brain may play a role in bone remodeling and disease. Therefore, the nervous system disorders can influence bone remodeling [30]. This is another possible mechanism by which cerebral atrophy may affect osteoporosis and cause OVCFs. However, this mechanism is also hypothetical and further human study will be necessary.

Our study has some limitations. First, due to the retrospective nature, our findings may be less accurate than those from a prospective study. We were not able to conduct a randomized control study, and retrospective studies are more likely to suffer from various types of bias. Moreover, its nature as a single-center study could limit the generalizability of our findings. Second, the number of patients was small because of the strict criteria for patient selection. Third, technical errors may have occurred during measurement of brain parenchyma and lateral ventricles volumes with the 3D slicer. Fourth, the interval between undergoing thoracolumbar radiographs and brain MRI was not consistent between all patients. And, we only included patients with thoracolumbar x-ray performed after the brain MRI. Fifth, our inclusion criteria of an osteoporotic T-score ≤ -2.5 is strict inclusion criteria. So, this restriction created a very selective group, and more likely to suffer from selection bias. However, data accuracy owing to consistent environmental conditions is a strength of a single-center study. Further study about brain degeneration and OVCF is needed to include not only the patients with T-score ≤ -2.5, but also the patients with T-score > -2.5. Lastly, the prevalence of cerebral atrophy could not be compared with other studies. Presence of cerebral atrophy was not measured dichotomously, but rather the volume of the brain using MRI. So it was impossible to measure the prevalence of cerebral atrophy.

The strength of our study is that this is the first to evaluate the brain volumes in patients with osteoporosis and OVCF. In summary, we observed a significant association between OVCF and volume percentage of brain parenchyma. We think that degeneration of the brain may lead to higher incidence of falling, and OVCFs may occur more frequently in patients with osteoporosis. We expect the findings of this study to expand our understanding of the association between brain degeneration and OVCF.
